# Transcriptomic analyses reveal clathrin-mediated endocytosis involved in symbiotic seed germination of *Gastrodia elata*

**DOI:** 10.1186/s40529-017-0185-7

**Published:** 2017-07-24

**Authors:** Xu Zeng, Yuanyuan Li, Hong Ling, Sisi Liu, Mengmeng Liu, Juan Chen, Shunxing Guo

**Affiliations:** Institute of Medicinal Plant Development, Chinese Academy of Medical Sciences & Peking Union Medical College, Beijing, 100193 People’s Republic of China

**Keywords:** *Gastrodia elata*, *Mycena*, Seed germination, Endocytosis

## Abstract

**Background:**

*Gastrodia elata* is a well-known medicinal orchid. In nature, the germination rate of *G. elata* is extremely poor, because there is no endosperm within the mature seed. It is crucial for *G. elata* to obtain nutrition from mycorrhizal fungi (*Mycena*) at the early-stage of germination. After germination, the seed gives rise to a protocorm. However, there are no “omic” studies on understanding the interaction between *Gastrodia* and *Mycena*. Here, we used transcriptomic approaches to explore changes in seed germination of *G. elata*.

**Results:**

Based on RNA-Seq, a total of ~221 million clean reads were assembled *denovo* into 139,756 unigenes, including 42,140 unigenes that were annotated in public databases. Meanwhile, 1750 unigenes were identified as differentially expressed genes. Most of these differentially expressed genes were putatively involved in energy metabolism, plant defense, molecular signaling, and secondary metabolism. Additionally, numerous genes involved in clathrin-mediated endocytosis were identified from our data. Most of these genes (e.g., clathrin, adaptor protein, dynamin, HSC70) were basally expressed in seeds and highly expressed in protocorms.

**Conclusions:**

Our data suggested that clathrin-mediated endocytosis could play important roles in symbiotic seed germination of *G. elata* with *Mycena* infections.

**Electronic supplementary material:**

The online version of this article (doi:10.1186/s40529-017-0185-7) contains supplementary material, which is available to authorized users.

## Background

In nature, the establishment of orchid seedlings requires compatible fungi to colonize the seed, thus providing nutrients for the formation of a protocorm. The protocorm is a post-embryonic structure from which both shoot and root systems subsequently differentiate. After the differentiation of green leaves, most orchid seedlings acquire autotrophy, while some orchids (known as fully mycoheterotrophic plants) are achlorophyllous and obtain their entire carbon source from their mycorrhizal fungi (Leake 2004; Dearnaley 2007).

*Gastrodia elata* is a fully mycoheterotrophic orchid that associates with two groups of fungal partners, i.e., *Mycena* and *Armillaria*, to complete its ontogenesis, including seed germination, tuber formation, flowering and fruiting. *Mycena* species (e.g., *M. dendrobii*) act as a symbiont during the stages of seed germination and protocorm development (Kim et al. 2006). For further development and enlargement of tubers, flowering and fruit setting, *Armillaria* becomes essential for the nutrient supply. It is also noteworthy that *G. elata* is a well-known Chinese medicinal orchid (Tsai et al. 2016). Recently, a number of pharmacological experiments indicated that it had strong potential to combat Alzheimer’s disease, Parkinson’s disease and other neurodegenerative diseases (Manavalan et al. 2012).

Plants are constantly exposed to a wide variety of microorganisms with either friendly or unfriendly intentions. Indeed, some plants are engaged in mutualistic or parasitic symbioses with them, or a plant-microorganism relationship, via a molecular dialogue. The plasma membrane (PM) of plants is a critical barrier that senses fungi and eventually allows their entry or the uptake of microbial molecules. Endocytosis is a process allowing extracellular particles or cargoes to enter the cell, whereby the PM invaginates and pinches off. In plants, Dhonukshe et al. (2007) have showed that clathrin-dependent endocytosis constitutes the predominant pathway for constitutive internalization of PM proteins.

In legume-*Rhizobium* symbiosis, the symbiotic relationship and nodule formation depend on mutual recognition based on a signal exchange between the two partners. Some studies revealed that *Medicago truncatula* root hair curling is associated with a strong stimulation of endocytosis at an early stage of symbiotic interaction with *Sinorhizobium meliloti*. Many plant genes associated with the initiation of endocytosis are induced, including Rab-like, Arf-like, and dynamin-like GTPases, and phosphatidylinositol 3-kinase. Most of these genes prevent root hair curling and infection thread formation (Leborgnecastel et al. 2010). However, the contribution of endocytosis to the establishment of orchid mycorrhizae has been hampered by a lack of data.

Although mycorrhizal fungi play a significant role in the symbiotic germination of orchids, the research of plant-fungi interactions in orchid mycorrhizae is very limited. In this study, we investigated changes in transcriptomic profiles during the symbiotic seed germination of *G. elata* inoculated with *M. dendrobii*. Meanwhile, endocytosis has been suggested to play a part in symbiosis between host-plant and endophyte (Wang et al. 2015). Therefore, we provided a detailed analysis of the expression patterns of genes involved in endocytosis.

Previous studies used green orchids as the experimental materials and presented some basic knowledge of the early plant-fungus interactions of orchid mycorrhizae. However, there is still no information about seed germination of achlorophyllous orchids. Since *G. elata* relies entirely on a nutrient supply from fungal partners, this model provides an ideal system to investigate the symbiotic seed germination of orchids with fungal infections. Our results have useful value for elucidating more information about the symbiotic seed germination of orchids with fungal infections.

## Methods

### Sample preparation

The flowers of *G. elata* were pollinated by hand in the production base at Shaanxi, China. Capsules were collected just prior to dehiscence. Meanwhile, the mycorrhizal fungal isolate (*M. dendrobii*) was incubated on fresh potato dextrose agar (PDA) in darkness at 25 °C. The procedure for symbiotic germination of *G. elata* was performed according to the description in a previous report (Kim et al. 2006). Germination performances were evaluated weekly under a stereo-microscope for 2 months. The protocorms were carefully collected under a stereo-microscope. For further transcriptomic studies, samples of equal fresh weight of *M. dendrobii* hyphae, *G. elata* seeds and protocorms were prepared and immediately stored at −80 °C.

### Histological study

The mycorrhizal protocorms were fixed in a solution of 2.5% glutaraldehyde and 1.6% paraformaldehyde in 0.1 M phosphate buffer (pH 6.8) overnight at 4 °C. The samples were washed with 0.1 M phosphate buffer, then dehydrated using an ethanol series, and embedded in Technovit 7100 (Kulzer & Co., Germany) as described by Yeung and Chan (2015). Then, 3 µm-thick sections were cut with glass knives using a Reichert-Jung 2040 Autocut rotary microtome. These sections were stained with periodic acid-Schiff reaction for total insoluble carbohydrates, and counterstained with either 0.05% (w/v) toluidine blue O for general histology or 1% (w/v) amido black 10B for protein. The sections were observed and the images were captured digitally using a CCD camera attached to a light microscope (AxioImager A1, Carl Zeiss AG).

### Total RNA extraction, library preparation, and transcriptome sequencing

Total RNA from each sample was extracted by using the RNeasy^®^ Plant Mini Kit (QIAGEN, GER) according to the manufacturer’s instructions. RNA degradation and contamination were checked on 1% agarose gels. In addition, the OD_260/230_ ratio and the RNA integrity number (RIN) were used for assessing RNA quality and purity using a Qubit^®^ 2.0 Fulorometer (Invitrogen, USA) and an Agilent 2100 Bioanalyzer system (Agilent Technologies, USA).

Library preparation and transcriptome sequencing were performed by the method previously described (Liu et al. 2015a). The cDNA library of each sample was prepared by using NEB Next^®^ Ultra™ RNA Library Prep Kit for Illumina^®^ (NEB, USA). Library quality was assessed on the Agilent 2100 Bioanalyzer system. After preparation of the library, an Illumina HiSeq 2000 platform was used for sequencing according to the manufacturer’s instructions and paired-end reads were obtained.

### Sequence read mapping, assembly, annotation and classification

All the bioinformatic analyses were performed according to a previous paper (Liu et al. 2015b). The data were cleaned by removing reads containing adapter, reads containing poly-N, and reads of low quality. Meanwhile, GC-content, Q20 and Q30 scores, and sequence duplication level were calculated. The clean reads were used in the following analyses.

As already known, it was technically impossible for us to remove the intracellular *M. dendrobii* hyphae from the *G. elata* protocorms. Unfortunately, *Mycena* and *Gastrodia* reference genomes have not yet been published now. Therefore, the hyphae group (*M. dendrobii*) was *denovo* assembled individually using Trinity with default parameters (kmer = 25) (Grabherr et al. 2011). The *Mycena* reference transcriptome was obtained. Then, the *M. dendrobii* reads derived from the protocorm group were removed by mapping all the reads against the *Mycena* reference transcriptome using Bowtie program v.0.12.8 (Langmead et al. 2009). Finally, the remaining unmapped non-BLAST and non-*Mycena* reads were prepared to detect the expression profiles of *G. elata*. All the clean reads from the seed and protocorm groups were assembled using Trinity (kmer = 25).

Distinct unigenes were indentified using the BLAST search program. All the unigenes were compared against the NCBI Nr/Nt, KOG, Swiss-Prot, KEGG and GO databases. The structure of mRNA sequences was much different between *Gastrodia* (a higher plant) and *Mycena* (fungus). Therefore, we have removed the unigenes that were not annotated with plant genes or annotated with fungus genes as reference. Finally, the remaining unigenes were used for functional analysis.

Prior to analyses of the differentially expressed genes (DEGs), the edgeR program package was used to adjust the read counts through one scaling normalized factor for each library. Then, the DEGseq (R package) was used for differential expression analysis of all libraries. The threshold for significantly differential expression was |log_2_(foldchange)| > 1 and P value <0.05 (Li and Dewey 2011).

Gene Ontology (GO) enrichment analyses were performed by the GOseq (R packages) based Wallenius non-central hyper-geometric distribution. The results were adjusted for gene length bias in DEGs (Young et al. 2010). In addition, WEGO (Web Gene Ontology Annotation Plot) were used for plotting GO annotation results (Ye et al. 2006). KEGG is a database for recoding the collection of high-level functions and the utility of the biological system. Here, KOBAS software was used for the statistical enrichment of DEGs in KEGG pathways (Kanehisa et al. 2008).

### Quantitative PCR

RNA from the same libraries was used for quantitative PCR analyses (Livak and Schmittgen 2001; Liu et al. 2015a). Primers designed with Primer Premier 5.0 are shown in Additional file 1: Table S1. PrimeScript™ RT reagent Kit (TaKaRa, JAP) was used for reverse transcription. In total, 1 μl of RT product diluted with 20 μl of ddH_2_O was used as template. Then, qPCR was performed in 15 μl of reaction mixture containing 7.5 μl of 2× SYBR^®^ Premix Ex Taq™ II (TaKaRa, JAP), 1.5 μl of cDNA template, and 0.3 μl of each gene-specific primer. In total, we performed two biological replicates and three technical replicates using the LightCycler^®^ 480 II RT-PCR System (Roche, SWIT) and its relative quantification software. The parameters of reactions were: 95 °C for 30 s, 40 cycles of 95 °C for 5 s, and 60 °C for 30 s. The cDNA libraries were standardized to reference gene 18S. The 2^−ΔΔCt^ method was used for evaluating gene expression.

## Results and discussion

### Development of mycorrhizal protocorms

**Fig. 1:**
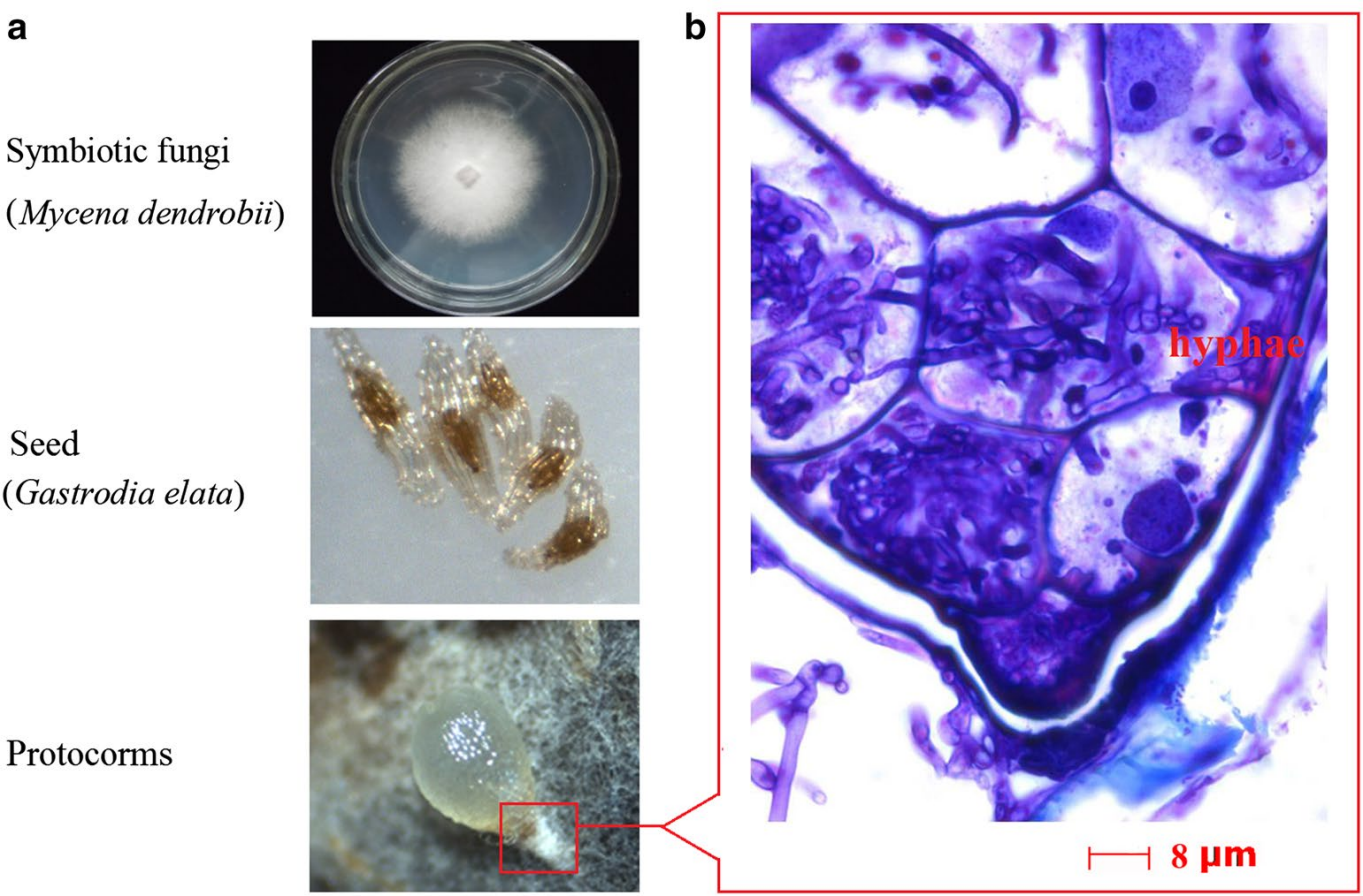
Biomorph of seed germination of *Gastrodia elata.* **a** *M. dendrobii* incubated on the fresh PDA; the mature seeds of *G. elata*; the typical protocorm of *G. elata* infected with *M. dendrobii*; **b** histological observation of the protocorms

The mature seed of *G. elata* contains a globular embryo covered by a thin layer of seed coat (Fig. 1a). By 4 weeks after inoculation, the seed was infected by fungal hyphae and had germinated. The embryo enlarged further and mycorrhizal protocorms formed. From histological observation, the cells in the basal region of the protocorm harbored a number of coiled intracellular fungal hyphae (the pelotons), while the shoot meristem was discernible at the apical region of the protocorm (Fig. 1b).

### Transcriptome profile

We performed RNA-Seq to comprehensively explore the crucial cluster of genes associated with symbiotic seed germination of *G. elata*. To increase the accuracies of the project, two biological replicates were included in each transcriptome sample of *G. elata* (seed and early-stage protocorm). Unfortunately, it was technically impossible for us to remove the intracellular fungal hyphae (*Mycena*) from the protocorms. This meant that symbiotic cells contained transcripts produced by both partners (*G. elata* and *M. dendrobii*). Therefore, RNA from a symbiotic fungi library was used for establishing a *Mycena* reference transcriptome. All of the *M. dendrobii* reads derived from the protocorm group were removed by mapping the reads against the *Mycena* reference transcriptome. Previous studies demonstrated that a bioinformatics pipeline could be effectively applied to assign genes identified in the transcriptome of plant-fungi association (Perotto et al. 2014; Liu et al. 2015a).

In our results, the sequencing error rate did not exceed 1% (Additional file 1: Table S2). In total, 235,192,510 raw reads and 221,982,110 clean reads were acquired from the seed and protocorm libraries. Since there were no reference genomes of *G. elata*, the clean reads from all libraries were pooled together and *denovo* assembled into transcripts. Finally, we obtained 179,781 transcripts with an N50 length of 1735 bp. We identified 139,756 unigenes with an N50 length of 1025 bp (length ranging from 201–17,061 bp). The datasets are available in the BioProject (Accession Number: PRJNA273548, mature seed and early-stage protocorm) repository of the NCBI.

### Gene annotation and function classification

Gene annotation was applied by BLAST against the NCBI Nr/Nt, Pfam, Swiss-Prot, GO, KOG and KEGG databases. In total, there were 42,140 unigenes (30.15% of all unigenes) annotated in at least one database (Table 1). Among them, a total of 30,090 unigenes (21.53%) were annotated in the Nr database. Based on Nr annotation, approximately 47.2% of annotated unigenes were assigned with a best score to a sequence from the top five species: *Elaeis guineensis*, *Musa acuminata*, *Phoenix dactylifera*, *Theobroma cacao* and *Vitis vinifera* (Additional file 1: Figure S1).

**Table 1:** Annotation result statistics between non-redundant unigenes and public databases

Database	Number of unigenes	Percentage (%)
Annotated in NR	30,090	21.53
Annotated in NT	13,503	9.66
Annotated in KO	9375	6.7
Annotated in SwissProt	22,893	16.38
Annotated in PFAM	24,874	17.79
Annotated in GO	25,105	17.96
Annotated in KOG	12,034	8.61
Annotated in all databases	3915	2.8
Annotated in at least one database	42,140	30.15
Total unigenes	139,756	100

According to our results shown in Additional file 1: Figure S2, 25,105 unigenes were annotated to 56 terms of GO classification. Among these terms, “cellular process” (13,851) and “metabolic process” (13,043) were dominant within “Biological Process” (BP). For “Cellular Component” (CC), dominant subcategories were “cell” (7548) and “cell part” (7542). In the category of “Molecular Function” (MF), “binding” (12,537) and “catalytic activity” (10,439) were highly represented. Moreover, the results showed that there were 211 and 3105 unigenes annotated to “immune system process” and “response to stimulus”, respectively. Similar results were reported by Zhao et al. (2013) and Perotto et al. (2014).

In Additional file 1: Figure S3, there were 12,034 unigenes annotated to 26 groups based on the KOG database. Among these groups, 2132 unigenes were annotated to “General Function Prediction Only”, 484 unigenes were annotated to “Carbohydrate metabolism and transport”, and 1389 unigenes were annotated to “Signal transduction mechanisms”. We also found that 84 unigenes were annotated to “Defence mechanisms”.

As shown in Additional file 1: Figure S4, all of the unigenes associated with “human diseases” were removed, and the remaining 9375 unigenes were mapped to 19 KEGG pathways. All the pathways were divided into five categories: “cellular processes”, “environmental information processing”, “genetic information processing”, “metabolism” and “organismal systems”. The pathways with the most representation were “translation” (1387 unigenes), “carbohydrate metabolism” (817 unigenes), and “Folding, sorting and degradation” (719 unigenes). In addition, there were 227 unigenes involved in the “signal transduction” pathways.

### Differential expression gene (DEG)

The normalized-FPKM (expected number of fragments per kilobase of transcript sequence per million base pairs sequenced) was used to quantify the transcript level in reads. The normalized-FPKM is a parameter for the comparison of mRNA levels among libraries. To increase the accuracies of the measured expression levels for further analyses, data from two biological replicates were merged, and FPKM values were calculated based on the merged dataset. The resulting Pearson’s correlation coefficients (R) were high between the replicates for seed (R = 0.659) and protocorm EP (R = 0.697) samples (Additional file 1: Figure S5).

**Fig. 2:**
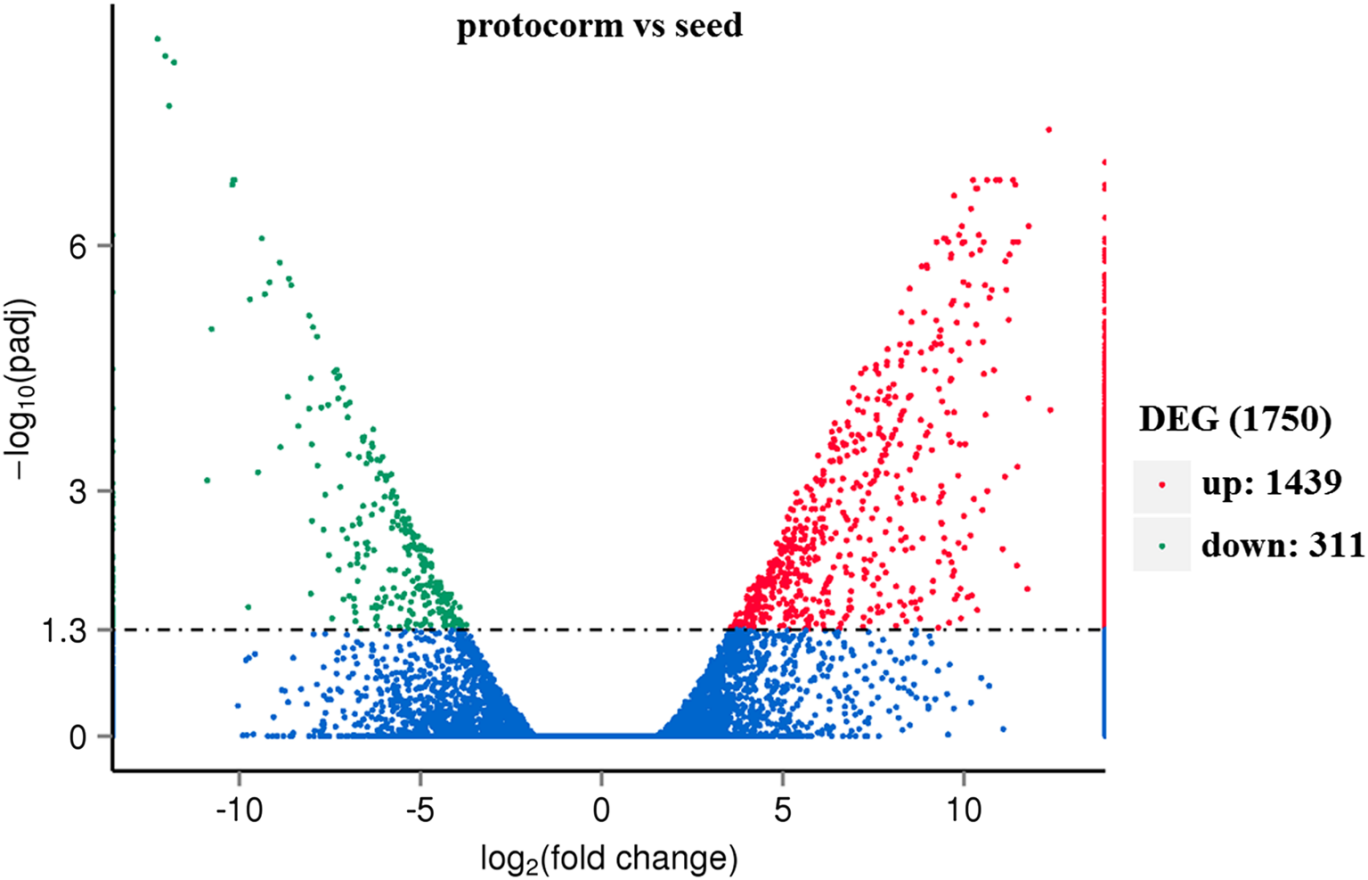
Volcano plots of differently expressed genes

**Fig. 3:**
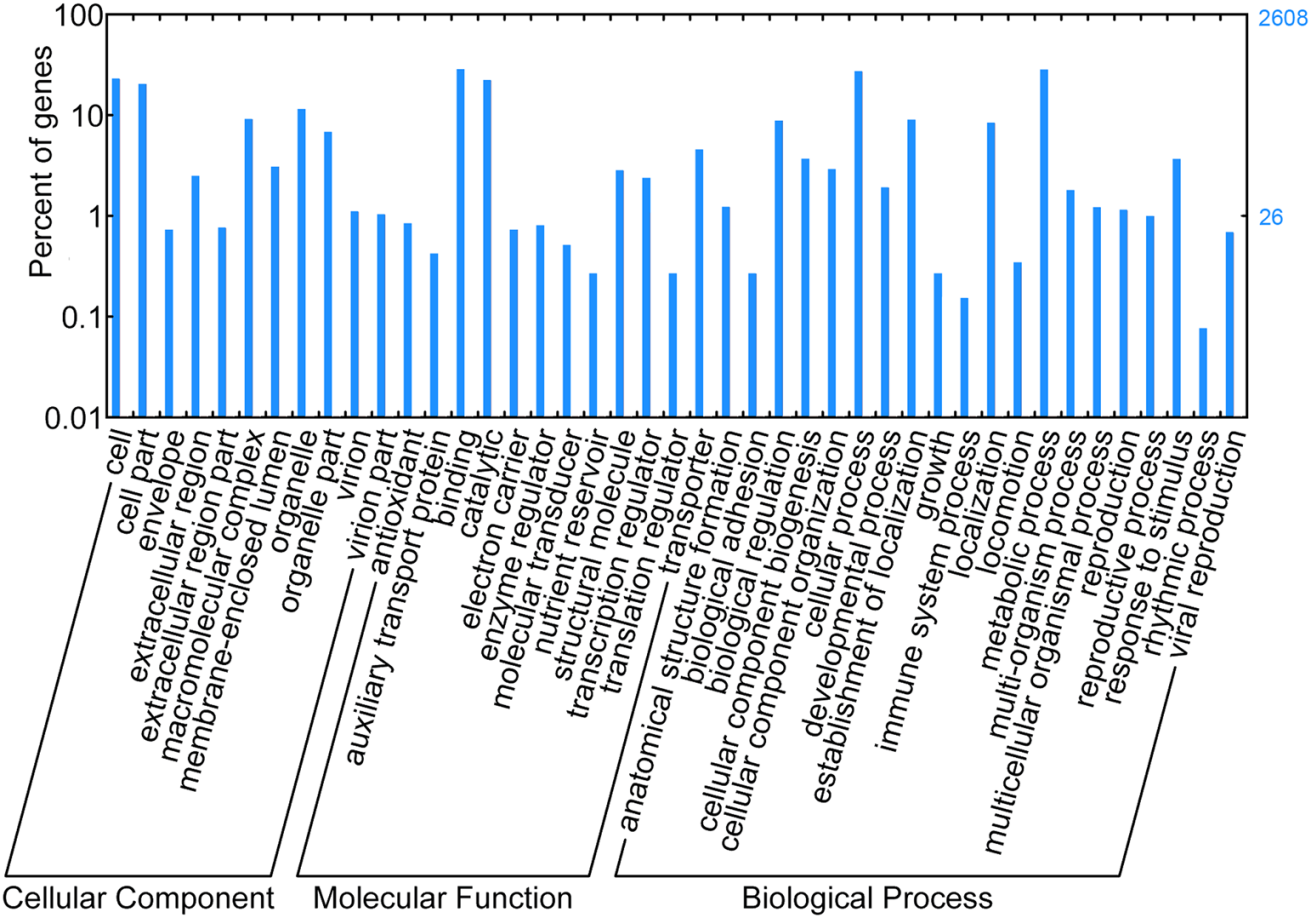
GO classification of differential expression genes (DEGs) in seeds vs. protocorms

**Fig. 4:**
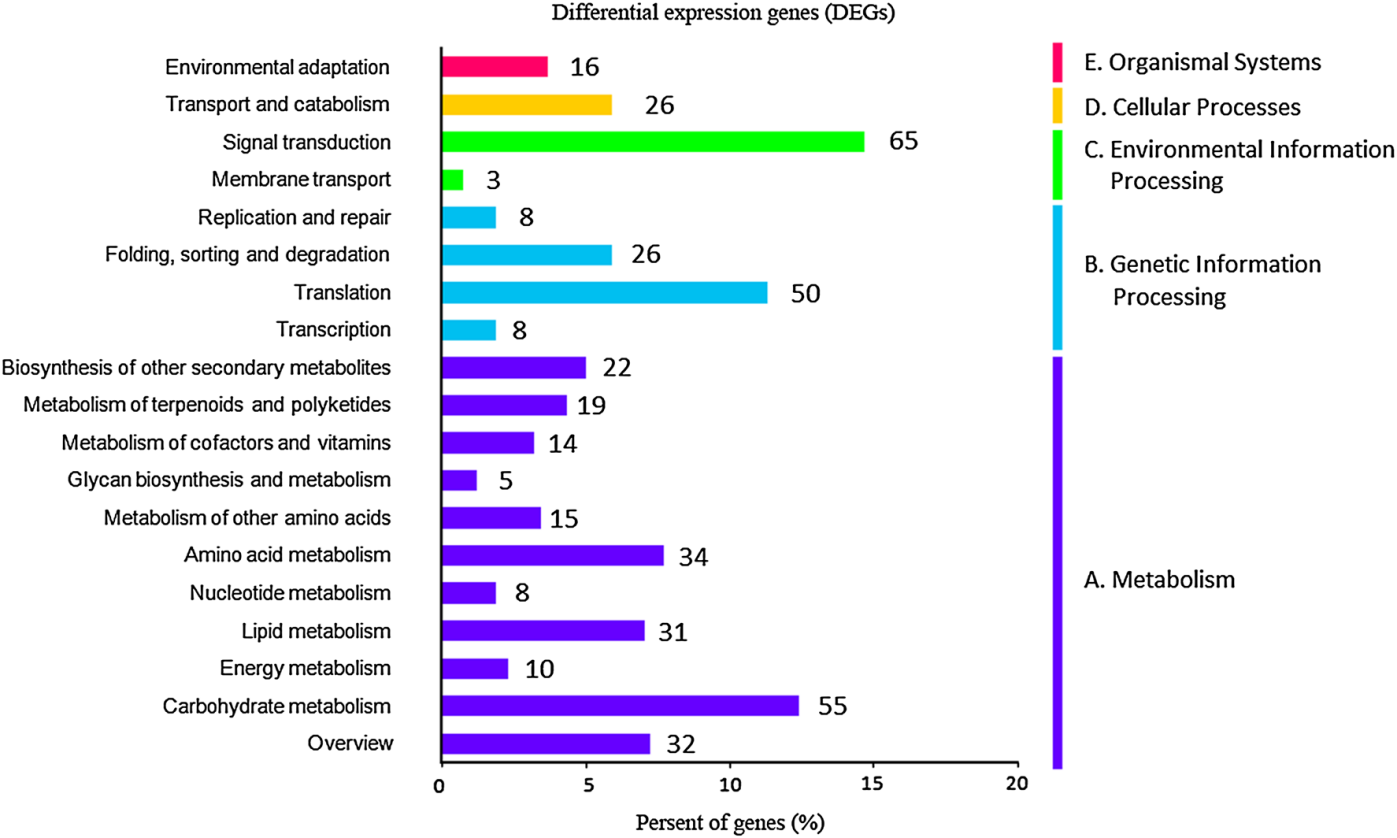
KEGG pathway enrichment scatter diagram of differential expression genes (DEGs) in seeds vs. protocorms

As shown in Fig. 2, differential expression genes (DEGs) were analyzed between the seeds and protocorms of *G. elata*. For seed vs. protocorm, 1750 unigenes were differentially expressed, including 1439 significantly up-regulated genes and 331 obviously down-regulated unigenes in the protocorm library. Based on GO enrichment (Fig. 3), DEGs were functionally classified according to the BP, CC, and MF categories and their subcategories. The largest subcategories for each functional group were as follows: “cellular process”, “metabolic process”, and “response to stimulus” for BP; “cell”, “cell part” and “organelle” for CC; “binding”, “catalytic”, and “structural molecule” for MF. According to KEGG (Fig. 4), most of the DEGs were assigned to “signal transduction”, “translation”, and “carbohydrate metabolism” pathways.

### Mutualistic symbiosis and endocytosis

In nature, orchid seeds possess no endosperm; therefore, they are devoid of nutrient supply. Mycorrhizal fungi provide the orchid seeds with signals and nutrients for germination, a mechanism called symbiotic germination. The process of orchid seed germination is unique among angiosperms and involves various processes such as fungi colonization, plant growth stimulation, plant response to fungi, etc. (Rasmussen 2002; Cameron et al. 2008; Rasmussen and Rasmussen 2009). In our subject, the transcriptomes of *G. elata* seeds and protocorms were sequenced. We selected 26 genes involved in clathrin-mediated endocytosis, including clathrin, adaptor protein complex (AP), AP180, adaptin-like protein, clathrin interactor epsin, dynamin-related protein (DRP), and heat shock cognate 70 kDa (HSC70). The expression of 16 genes was significantly up-regulated in protocorms compared with seeds, according to RNA-Seq results. Our results suggested that clathrin-mediated endocytosis could play an important role in the symbiotic seed germination of orchids with fungal infections.

Endocytic mechanisms are essential for regulation of lipid and protein compositions of the plasma membrane. A previous study showed that the establishment of endocytosis was a crucial process involved in many diverse aspects of plant life, such as basic cellular functions, growth development, and hormonal signaling (Fan et al. 2015). Another important role of plant endocytosis is in communication with the environment, including nutrient uptake, toxin avoidance, and pathogen defense. Endocytosis in plants is essential for the acquisition of materials from the extracellular space. At the moment, endocytosis is generally divided into clathrin-mediated endocytosis and clathrin-independent endocytosis. The majority of endocytosis in plants depends on the coat protein clathrin (Chen et al. 2011).

A previous study showed that endocytosis played a role in symbiosis between legumes and rhizobia (Wang et al. 2015). A complex molecular mechanism was required for the establishment of symbiotic relationships between legumes and rhizobia. Legume roots released flavonoids, which triggered synthesis and secretion of rhizobial nodulation factors. These nodulation factors were recognized by legume roots and activated a symbiosis signaling pathway. The legumes allowed the rhizobia to enter root hairs and pass through plant-derived infection threads. Finally, rhizobia were released from infection threads (IT) to symbiosomes of infected cells within the root nodule. A previous study suggested that the nodulation factor signaling pathway might proceed through an endocytosis process (Timmers et al. 1998). Another study found that the *Medicago* flotillin-like proteins, lipid raft-associated integral membrane proteins involved in endocytosis, were crucial for infection thread initiation and nodule development (Haney and Long 2010).

In legume-*Rhizobium* symbiosis, a nod factor signaling pathway may proceed via ligand-induced receptor endocytosis, similar to that characterized in plant defense against pathogens. Nod factors are recognized by receptors of the LysM domain-containing receptor-like kinase family at the PM and have been detected inside root hairs, along the IT, and in the cytoplasm of infected root cells (Leborgnecastel et al. 2010).

Surface polysaccharides (SPS) from rhizobia could also be likely substrates for uptake by endocytosis. SPS are crucial signaling molecules for the development of rhizobial infection and are thought to attenuate or suppress defense reactions. Internalization of SPS has been observed in plant cells (Gross et al. 2005), thus substantiating the existence of specialized receptors, as previously proposed for legume-*Rhizobium* symbiosis.

### Clathrin-mediated endocytosis

**Fig. 5:**
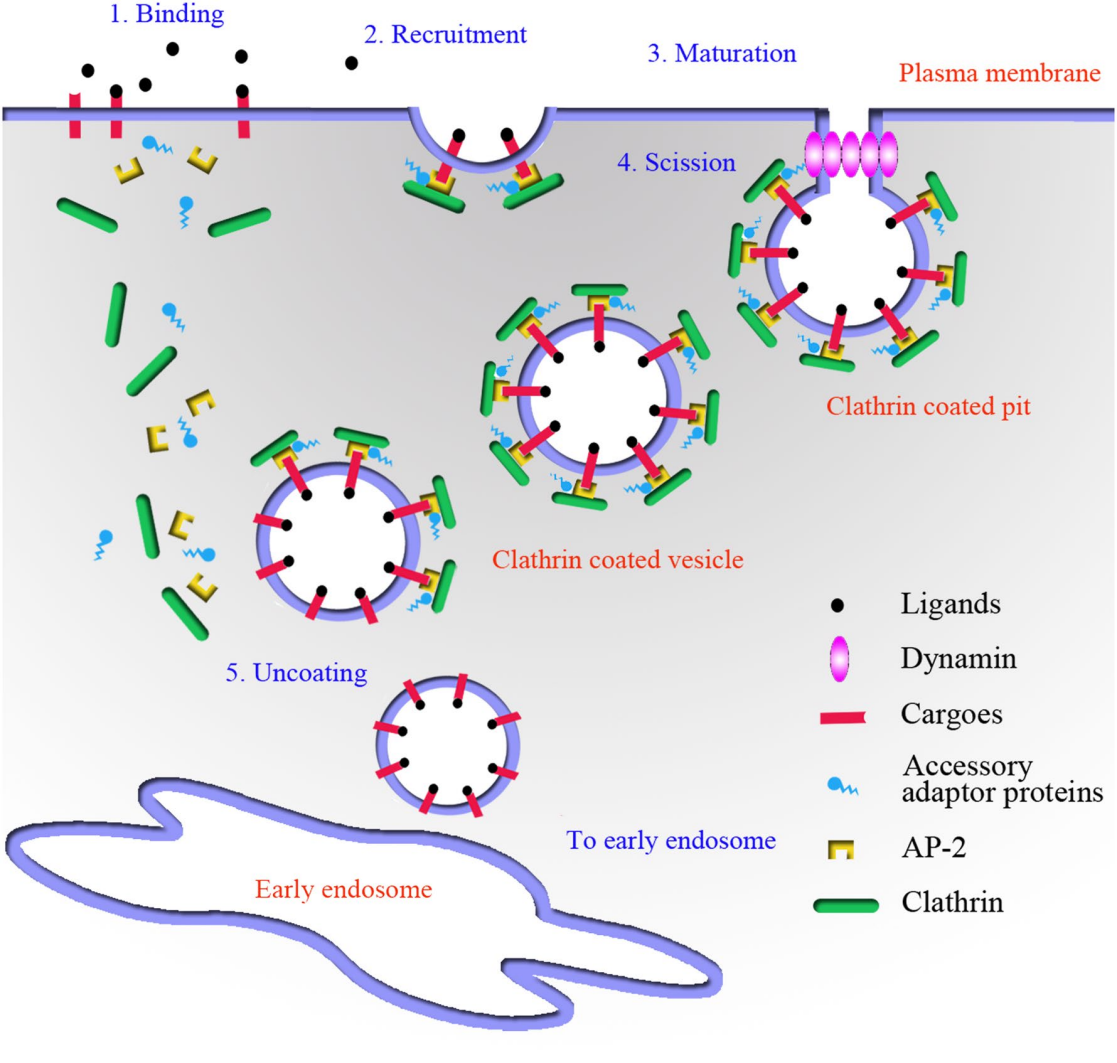
Quantitative PCR analysis of the putative genes

As shown in Fig. 5, prior observations have defined the five steps of clathrin-coated vesicle formation (Doherty and McMahon 2009; McMahon and Boucrot 2011). Clathrin-mediated endocytosis initiates at the plasma membrane with the recruitment of cargo. Cargoes that need to be internalized from the plasma membrane are packaged by co-operative assembly of coat proteins, including clathrin, adaptor protein complex and associated accessory adaptor proteins (adaptin, intersectin, AP180, clathrin interactor epsin, TPLATE-like protein, etc.). Clathrin-coated pits (CCPs) then mature, and dynamin and DRPs assemble at the neck of the bud for scission of the CCPs from the plasma membrane. Once detached from the membrane and formed into clathrin-coated vesicles (CCVs), the clathrin coat disassembles in seconds to form uncoated vesicles that fuse with the early endosome (EE), where the cargo is further sorted. ATPase HSC70 and its cofactors (auxilin, etc.) are necessary for uncoating of CCVs and clathrin component recycling.

In our RNA-Seq results (Table 2), 16 genes involved in clathrin-mediated endocytosis were significantly up-regulated in protocorms compared with those in seeds, including clathrin, adaptor protein, adaptin-like protein, A180, epsin, DRP and HSC70. These genes involved in clathrin-mediated endocytosis may therefore be associated with the symbiotic seed germination of orchids with fungal infections. In agreement with our results based on the comparative transcriptome analysis of symbiotic and asymbiotic seed germination of *Anoectochilus roxburghii* (NCBI Accession Number: PRJNA299493), we also found that many genes involved in clathrin-mediated endocytosis (e.g., clathrin, epsin, dynamin, HSC70, AP180) were significantly up-regulated in symbiotic protocorms compared with those in asymbiotic protocorms.

**Table 2:** The up- and down-regulated genes in protocorms of *G. elata* compared with seeds

Unigene ID	Putative gene	Refseq	Ratio change
c113523_g1	CHC	XP_010907410.1	2.56*
c113909_g1	CLC	XP_008802347.1	1.89
c29883_g1	AP-1 β	XP_009385856.1	1.92
c36857_g1	AP-1 μ	XP_010923038.1	1.28
c49102_g1	AP-1 σ	AFS50008.1	2.26*
c98691_g1	AP-1 γ	XP_010913560.1	2.31*
c29046_g1	AP-2 μ	XP_007039526.1	1.68
c33125_g1	AP-2 α	XP_010933729.1	2.59*
c126705_g1	AP-3 δ	XP_009396369.1	0.9
c29490_g1	AP-3 σ	XP_010939294.1	1.54
c46833_g1	AP-3 β	XP_010937611.1	0.95
c50113_g1	AP-3 μ	XP_010921397.1	1.17
c43068_g1	AP-4 ε	XP_010909540.1	2.09*
c50920_g1	AP-4 σ	XP_007152949.1	2.39*
c28875_g1	Adaptin	XP_008792344.1	2.40*
c50842_g1	AP180	XP_009396332.1	2.18*
c36930_g2	Epsin 1	XP_008799757.1	2.04*
c50396_g1	Epsin 2	XP_009392171.1	1.62
c45022_g1	Epsin 3	XP_010922341.1	0.96
c29413_g1	DRP3	XP_008794571.1	3.07*
c32547_g1	DRP5	XP_010918171.1	3.84*
c43852_g2	DRP1	XP_010933528.1	5.75*
c56183_g1	DRP2	XP_008779526.1	2.48*
c29657_g1	HSC70	XP_011094842.1	2.08*
c33306_g1	HSC70	XM_009382388.1	2.45*
c7920_g1	HSC70	XP_011027182.1	2.23*

Only differential expression genes with change ratios ≥2 (up) or ≤0.5 (down) were marked with asterisk (*). Transcriptomic analyses were performed on the seeds and protocorms of *G. elata*. Of the 26 genes involved in clathrin-mediated endocytosis, 16 genes were found to be significantly up-regulated in the protocorms based on RNA-seq analyses. *CHC* clathrin heavy chain, *CLC* clathrin light chain, *AP-1 β/μ/σ/γ* adaptor protein-1 complex subunit beta/mu/sigma/gamma, *AP-2 α/μ* adaptor protein-2 complex subunit alpha/mu, *AP-3 δ/σ/β/μ* adaptor protein-3 complex subunit delta/sigma/beta/mu, *AP-4 ε/σ* adaptor protein-4 complex subunit epsilon/sigma, *AP180* clathrin assembly protein, *espin* clathrin interactor epsin, *DRP* dynamin-related protein, *HSC70* heat shock cognate 70 kDa

### Clathrin

Clathrin plays an important role in many cellular processes, such as endosomal sorting, protein secretion, mitosis, and endocytosis. Since the subunits are recruited from the cytoplasm, clathrins form three-legged molecules, termed hexameric triskelions. In general, clathrin is composed of three heavy chains (CHCs) and three light chains (CLCs). Two CHC genes and three CLC genes exist in the *Arabidopsis* genome (Fan et al. 2015). Our results (Table 2), show that one CHC gene and one CLC gene were expressed during seed germination of *G. elata* infected with *M. dendrobii*. Of these genes, the CHC gene (c113523_g1) was up-regulated in protocorms compared to that in seeds.

### Adaptor complexes

Clathrin is not able to directly bind membranes or cargoes. At the budding site, adaptor protein complexes are recruited for linking lipids, cargo, and adaptor proteins to the clathrin triskelion. Of the four AP complexes (AP1-4), the highly conserved AP2, which comprises two large (α, β) subunits, a medium (μ) and a small (σ) subunit, serves as a central player in the initiation of CCP formation. A previous study showed that orthologs of different adaptins were present in plant genomes, including AP2 components (Chen, Irani et al. 2011). Here, we found that 12 genes encoding AP1 (β/μ/σ/γ), AP2 (α/μ), AP3 (δ/σ/β/μ) and AP4 (ε/σ) were expressed during symbiotic seed germination. Among these genes, AP1 σ and γ (c49102_g1 and c98691_g1), AP2 α (c33125_g1), and AP4 ε (c43068_g1) genes were differentially up-regulated on transcriptional levels in protocorms compared with those in seeds (Table 2).

In addition to the classical adaptins, a large number of accessory proteins have a similar function in linking cargo and lipids to the AP2-clathrin assembly, including epsin, AP180, eps15, and TPLATE. These accessory proteins were also able to influence actin dynamics and induce membrane curvature for invagination of the plasma membrane into vesicles (Fan et al. 2015). In our study, three epsin (1/2/3) genes, one AP180 gene, and one adaptin-like gene were observed in our data. Furthermore, three accessory proteins, epsin 1, AP180 and adaptin-like protein (c36930_g2, c50842_g1 and c28875_g1) showed increased accumulation in protocorms according to transcriptomic data.

### Dynamin

Following maturation of CCPs, dynamins are recruited by BAR domain-containing proteins at the CCP neck. As membrane scission proteins, dynamins are members of a large family of GTPase proteins that form a helical polymer around the constricted CCP neck and mediate fission of the vesicle from the plasma membrane, thus releasing the CCV into the cell. Of the six dynamin-related protein families in plants, DRP1 and DRP2 were demonstrated to be involved in clathrin-mediated membrane trafficking (Chen et al. 2011; McMahon and Boucrot 2011). In total, four dynamin-related genes were identified in our results (Table 2). Our study also revealed that four dynamin-related proteins, DRP1, 2, 3 and 5, were differentially up-regulated on transcriptional levels in protocorms compared with those in seeds.

### HSC70

Once detached from the plasma membrane, the clathrin coat is subsequently released from the CCVs by ATPase HSC70 and its cofactor, auxilin (or cyclin G-associated kinase in non-neuronal tissues). The uncoated CCVs then undergo further trafficking within the cell and fuse with early endosomes. According to our data (Table 2), three HSC70 genes were observed during symbiotic seed germination. All of these genes (c29657_g1, c33306_g1 and c7920_g1) were differentially up-regulated on transcriptional levels in protocorms compared with those in seeds.

### qPCR analysis of putative genes

**Fig. 6:**
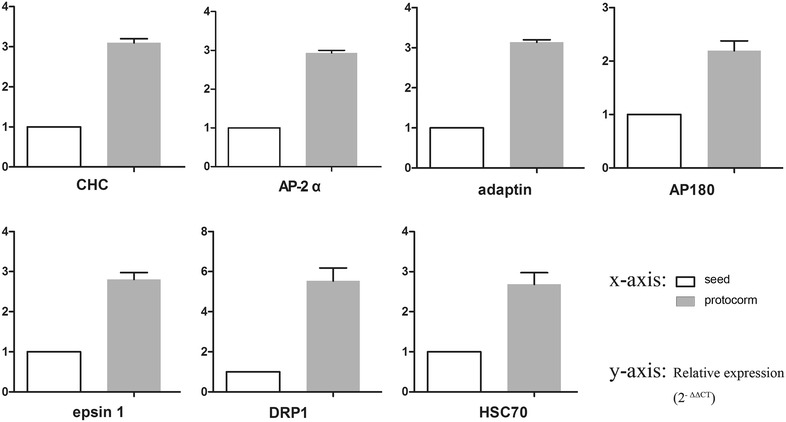
Clathrin-mediated endocytosis

To confirm the reliability of the RNA-Seq data, we performed quantitative PCR analysis on 7 selected genes putatively involved in the endocytosis mechanism, such as clathrin (c113523_g1), adaptor protein (c33125_g1), epsin (c36930_g2), adaptin-like protein (c28875_g1), AP180 (c50842_g1), dynamin-related protein (c43852_g2) and heat shock cognate 70 kDa (c29657_g1). The results of qPCR related to expression changes of these genes are shown in Fig. 6. In our results, most of the genes showed low expression levels in seeds and high expression levels in protocorms during the developmental process of seed germination in *G. elata*. Taken together, all the unigenes showed expression patterns that were consistent with the transcriptomic data, indicating that our experimental results were valid.

## Conclusions

In conclusion, transcriptomic analyses provide a powerful method for investigating putative genes involved in the symbiotic seed germination of orchids. In this study, we performed transcriptome sequencing of mature seeds and early-stage protocorms from *G. elata* to identify these genes and quantify their expression in seed germination. Subsequently, we analyzed putative differently expressed genes of clathrin, adaptor protein, AP180, adaptin-like protein, clathrin interactor epsin, dynamin-related protein, and HSC70 that were produced in the process of plant endocytosis. Our results indicated that the up-regulation of expression of genes related to clathrin-mediated endocytosis could play an important role in symbiotic germination in *G. elata* infected by *M. dendrobii*, especially at the early stage of protocorm development. The RNA-Seq data from our study provide an important resource for studying interactions between plant seeds and symbiotic fungi. Further genomic research of orchids and mycorrhizal fungi will provide new insights into the interactions between fungi and plants.

## Additional file

**Additional file 1: Figure S1.** Characteristics of similarity search of unigenes against NR database. **Figure S2.** GO classification of all unigenes from RNA-Seq. **Figure S3.** KOG classification of all unigenes from RNA-Seq. **Figure S4.** KEGG pathway enrichment scatter diagram of all unigenes from RNA-Seq. **Figure S5.** Correlation of RPKM distribution between two biological replicates. **Table S1.** The quantitative PCR primers of putative genes. **Table S2.** Statistical summary of sequences analysis. Q20 and Q30: The percentage of bases with a Phred value > 20 and 30.
