# Supplementary material for: Transcriptomic analyses reveal clathrin-mediated endocytosis involved in symbiotic seed germination of Gastrodia elata
Source: Bot Stud. 2017 Jul 24;58:31. doi: 10.1186/s40529-017-0185-7 (PMC5524656; doi:10.1186/s40529-017-0185-7)
Supplement: Supplementary file 1 — Additional file 1: Figure S1. Characteristics of similarity search of unigenes against NR database. Figure S2. GO classification of all unigenes from RNA-Seq. Figure S3. KOG classification of all unigenes from RNA-Seq. Figure S4. KEGG pathway enrichment scatter diagram of all unigenes from RNA-Seq. Figure S5. Correlation of RPKM distribution between two biological replicates. Table S1. The quantitative PCR primers of putative genes. Table S2. Statistical summary of sequences analysis. Q20 and Q30: The percentage of bases with a Phred value > 20 and 30. [file 40529_2017_185_MOESM1_ESM.docx]

**Transcriptomic analyses reveal clathrin-mediated endocytosis involved in symbiotic seed germination of *Gastrodia elata***

**Xu Zeng^+^, Yuanyuan Li****^+^, Hong Ling, Sisi Liu, Mengmeng Liu, Juan Chen* and Shunxing Guo*******

Institute of Medicinal Plant Development, Chinese Academy of Medical Sciences, Beijing, 100193, P.R. China

***Corresponding:** kibchenjuan@126.com; [sxguo2015@163.com](mailto:sxguo2015@163.com)

**^+^** These authors contributed equally to this work

**Supplementary information**

**Figure S1.** Characteristics of similarity search of unigenes against NR database.

**Figure** **S2.** GO classification of all unigenes from RNA-Seq.

**Figure S3.** KOG classification of all unigenes from RNA-Seq.

**Figure S4.** KEGG pathway enrichment scatter diagram of all unigenes from RNA-Seq.

**Figure S5.** Correlation of RPKM distribution between two biological replicates.

**Table S1.** The quantitative PCR primers of putative genes.

**Table S2.** Statistical summary of sequences analysis. Q20 and Q30: The percentage of bases with a Phred value > 20 and 30.


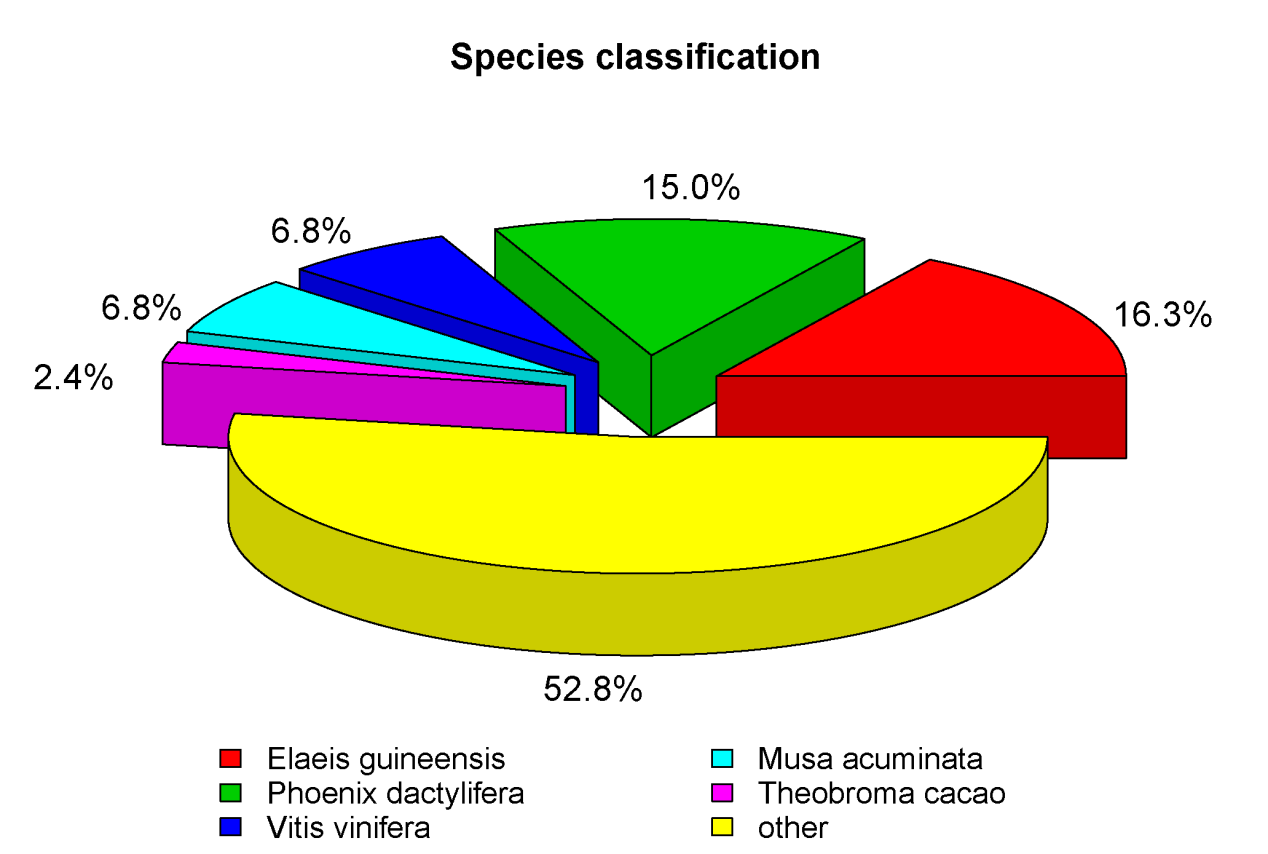


**Figure S1. Characteristics of similarity search of unigenes against NR database.**


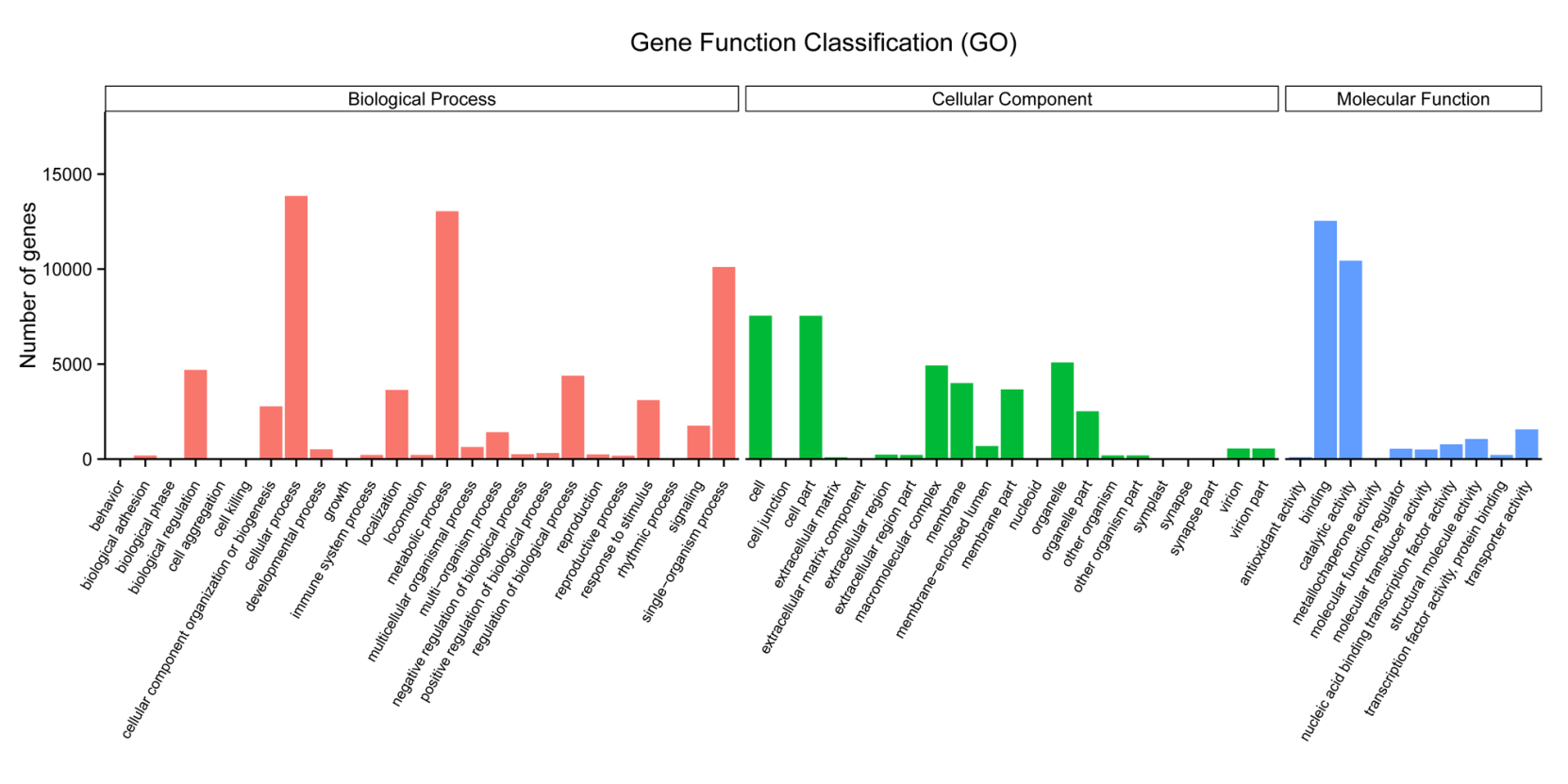


**Figure** **S2.** **GO classification of all unigenes from RNA-Seq.**


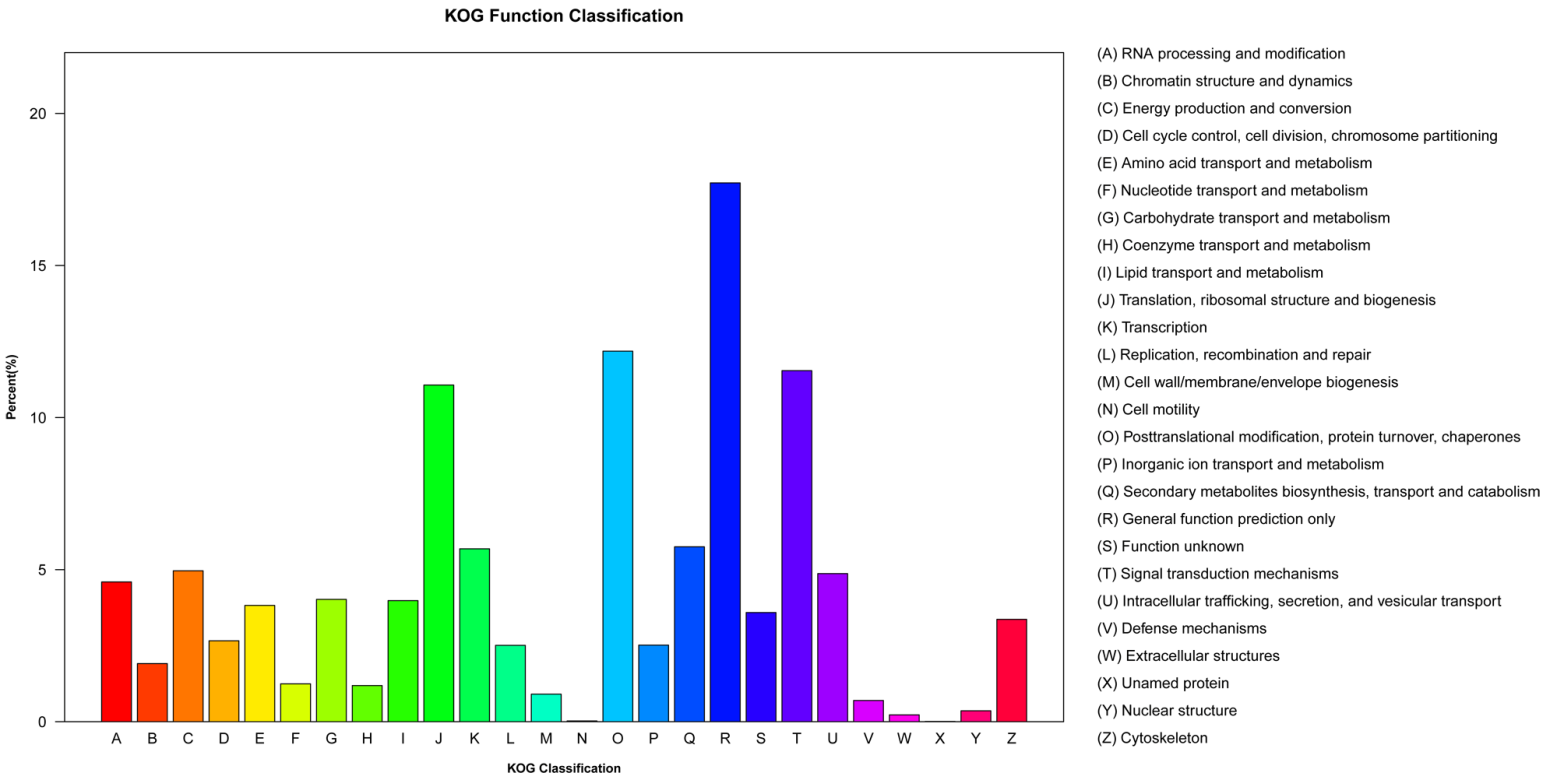


**Figure S3.** **KOG classification of all unigenes from RNA-Seq.**

**
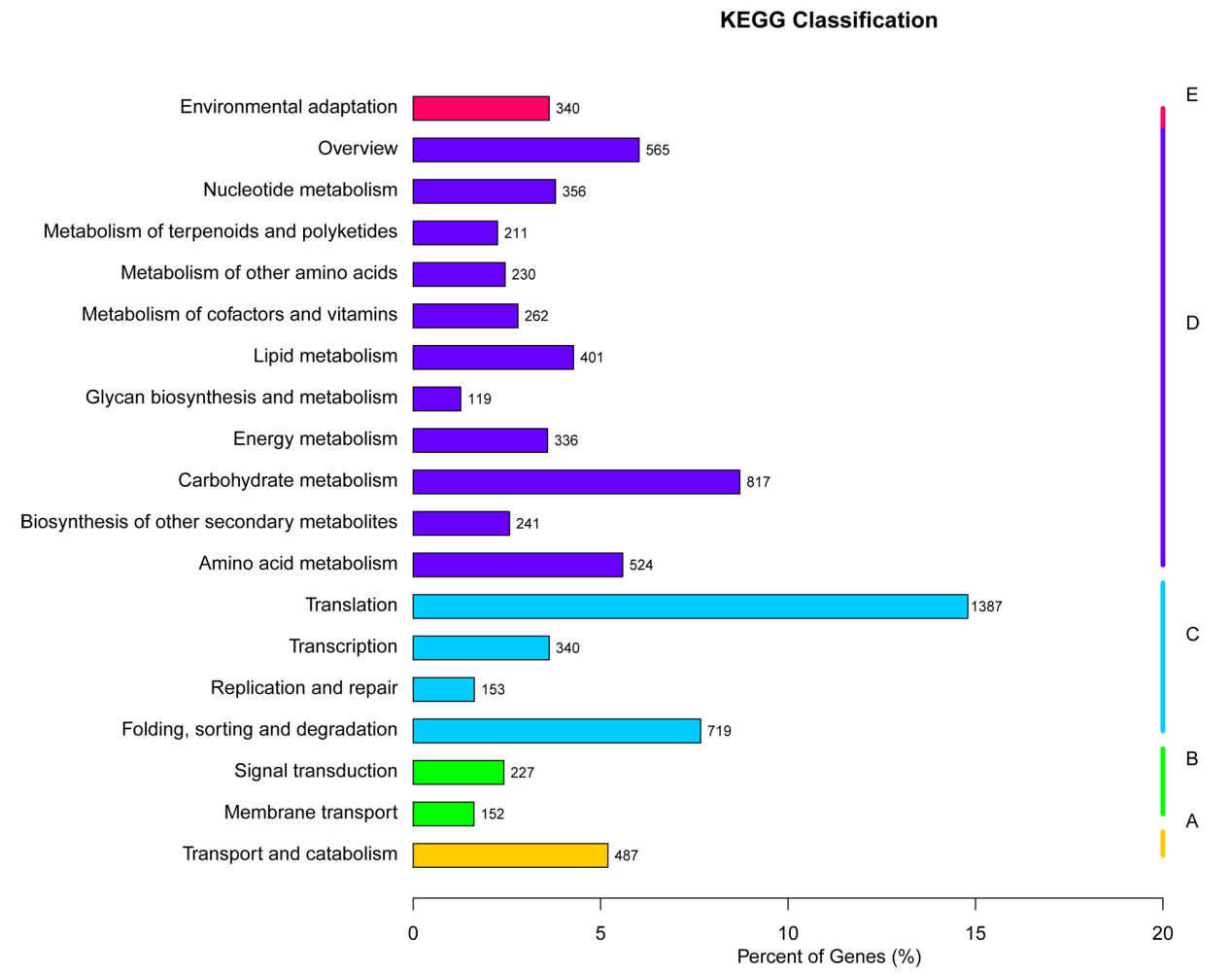
**

**Figure S4. KEGG pathway enrichment scatter diagram of all unigenes from RNA-Seq.**

**
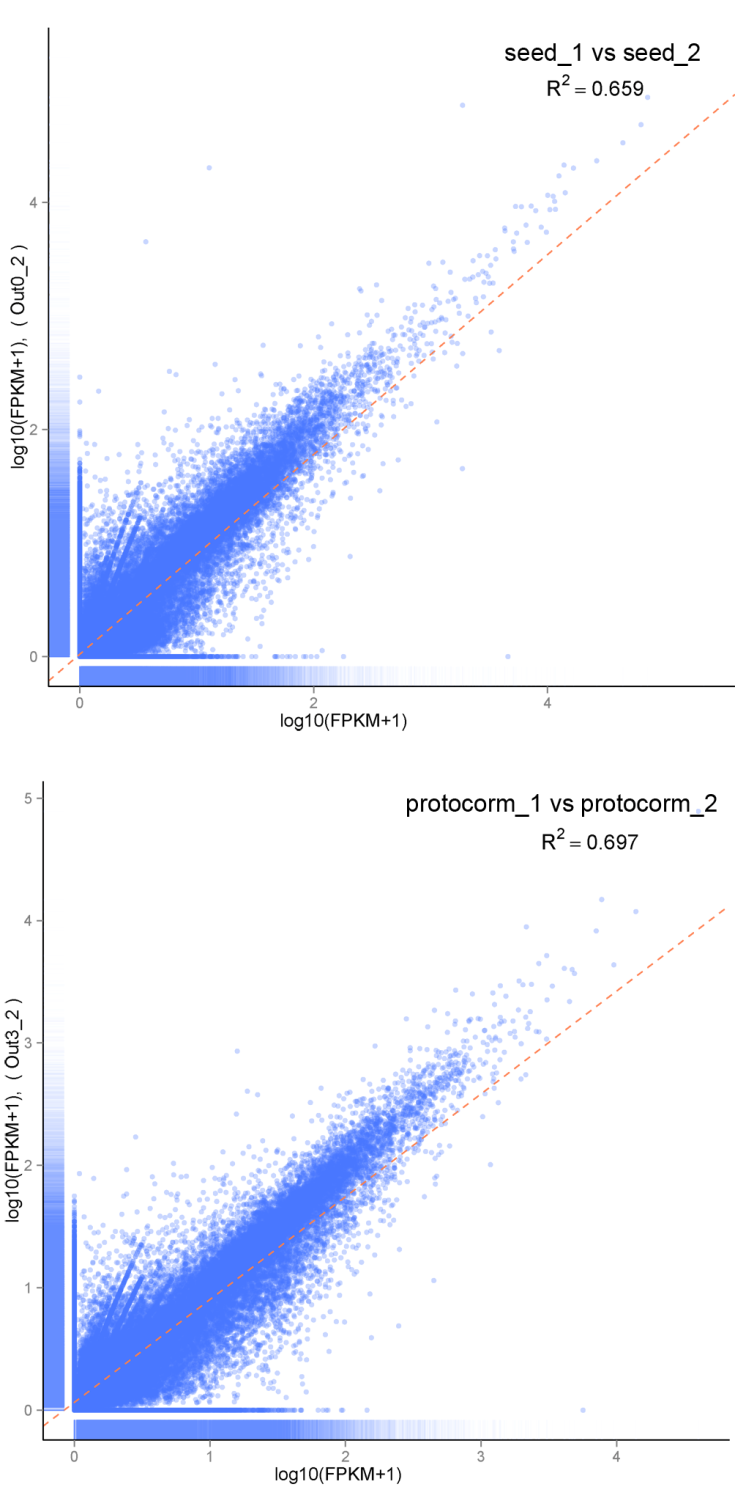
**

**Figure S5.** Correlation of RPKM distribution between two biological replicates.

**Table S1.** The quantitative PCR primers of putative genes.

| **Unigene ID** | **Forward primer(5'-3')** | **Reverse primer(5'-3')** |
| --- | --- | --- |
| 18S rRNA | CCAGGTCCAGACATAGTAAG | GTACAAAGGGCAGGGACGTA |
| c113523_g1 | TCAAGGAGCCTAAGGTGGACAGT | GCATCAACACAAGCGAAGCAGAC |
| c33125_g1 | CAGGAAGTGGTTAGAGGCGTTAAGC | CGGAAGCAATGGTAAGGCGAAGT |
| c36930_g2 | AAGTTGATGGATCGGATGACCTCTT | GCTGGCTGGTTGGCACAGATAT |
| c28875_g1 | TGCCAATACAGTGAGCCATCGT | GGTCTCCGCAGTCACATAATCCTT |
| c50842_g1 | AGACGGCTGGCTAAGACTCACAA | AAGCAGAGCAATCCCAAGCAATAGG |
| c43852_g2 | GAAGCCGTCCACTTGGTTATGAAGG | CCGCCGCTAATTCCGACTGAAG |
| c29657_g1 | CAACCGCACCACGCCATCTT | GTGTATCGCCTGCCAATCAATCTCT |

**Table S2.** Statistical summary of sequences analysis. Q20 and Q30: The percentage of bases with a Phred value > 20 and 30.

| **Sample** | **Raw Reads** | **Clean Reads** | **Clean Bases** | **Error**  **(%)** | **Q20(%)** | **Q30(%)** | **GC (%)** |
| --- | --- | --- | --- | --- | --- | --- | --- |
| seed1_1 | 33805127 | 31355902 | 3.14G | 0.03 | 97.09 | 91.57 | 49.03 |
| seed1_2 | 33805127 | 31355902 | 3.14G | 0.02 | 96.29 | 91.42 | 51.2 |
| seed2_1 | 23848399 | 22596255 | 2.26G | 0.04 | 97.03 | 91.26 | 48.98 |
| seed2_2 | 23848399 | 22596255 | 2.26G | 0.04 | 96.17 | 89.83 | 49.03 |
| protocorm1_1 | 36158168 | 34556037 | 3.46G | 0.03 | 97.79 | 92.75 | 47.64 |
| protocorm1_2 | 36158168 | 34556037 | 3.46G | 0.03 | 96.86 | 90.97 | 47.64 |
| protocorm2_1 | 23784561 | 22482861 | 2.25G | 0.03 | 97.66 | 92.35 | 47.61 |
| protocorm2_2 | 23784561 | 22482861 | 2.25G | 0.02 | 96.66 | 91.96 | 48.66 |
| Total | 235192510 | 221982110 | 22.22G |  |  |  |  |
